# N4-Acetyl-Sulfamethoxazole Bladder Stones Caused by Trimethoprim-Sulfamethoxazole Prophylaxis: A Report of Two Cases

**DOI:** 10.7759/cureus.111985

**Published:** 2026-07-03

**Authors:** Emma M Waddell, Marie-Therese Valovska, Daniel A Wollin, Martin Kathrins

**Affiliations:** 1 Urology, Brigham and Women's Hospital, Harvard Medical School, Boston, USA; 2 Urology, University of Washington, Seattle, USA

**Keywords:** benign prostatic hyperplasia, bladder stones, case report, cystolitholapaxy, drug-induced urolithiasis, n4-acetyl-sulfamethoxazole, trimethoprim-sulfamethoxazole

## Abstract

The combination antimicrobial trimethoprim-sulfamethoxazole (TMP-SMX) has a broad spectrum of activity and is used as a prophylactic for various infections. Drug-induced urolithiasis is a rare side effect of TMP-SMX that results from the precipitation of its metabolite N4-acetyl-sulfamethoxazole. There are only two reports of bladder stones associated with TMP-SMX, both of which occurred in patients with a history of recurrent urinary tract infections (UTIs).

At our institution, an 80-year-old male with a complex medical history including benign prostatic hyperplasia (BPH) and a chronic prosthetic joint infection on long-term amoxicillin and TMP-SMX presented for evaluation of an incidental 2.4 cm bladder stone. Surgical intervention was initially deferred due to patient preference, but he later developed gross hematuria and decided to undergo cystoscopy with cystolitholapaxy and bilateral retrograde pyelogram. The procedure was completed without complications. Analysis of stone fragments revealed that they were 100% N4-acetyl-sulfamethoxazole. Months later, a 70-year-old male with multiple sclerosis and neurogenic bladder complicated by recurrent UTIs managed with a suprapubic catheter and TMP-SMX prophylaxis was found to have multiple bladder stones causing obstructive symptoms. These stones were similarly treated with cystolitholapaxy and identified as 100% N4-acetyl-sulfamethoxazole.

Bladder stones caused by TMP-SMX have been rarely reported in people like our 70-year-old patient with an abnormal urinary metabolic profile, indicated by a history of urolithiasis or UTIs. Our other patient did not have either of these risk factors, but he did have lower urinary tract symptoms due to BPH. His case may serve as a caution to the use of TMP-SMX in the setting of chronic urinary stasis due to bladder outlet obstruction.

## Introduction

Trimethoprim-sulfamethoxazole (TMP-SMX) is a bactericidal antimicrobial commonly used to treat urinary tract infections, shigellosis, salmonellosis, and Pneumocystis jirovecii pneumonia. In orthopedics, TMP-SMX has emerged as an effective salvage therapy for polymicrobial bone and joint infections, even in the setting of a prosthesis [1]. Oral TMP-SMX demonstrates good bone diffusion with coverage against Gram-positive and Gram-negative bacteria, including methicillin-resistant *Staphylococcus aureus* [1].

The most frequent side effects of TMP-SMX are gastrointestinal upset and cutaneous reactions. Nephrotoxicity associated with TMP-SMX is rare, but mechanisms include interstitial nephritis, acute tubular injury, and intra-tubular obstruction or crystallization of the SMX component. There are very few case reports of drug-induced urolithiasis due to TMP-SMX, only one of which describes bladder stones in the absence of concomitant renal or ureteral stones [2]. Here, we present two patients on long-term TMP-SMX with no history of urolithiasis who developed isolated bladder stones composed entirely of the TMP-SMX metabolite, N4-acetyl-sulfamethoxazole.

## Case presentation

Case 1

The first patient was an 80-year-old male with a urological history of benign prostatic hyperplasia (BPH) treated with transurethral resection of prostate (TURP) seven years earlier, who presented for evaluation of a 2.4 cm bladder stone incidentally identified on a CT scan of the abdomen and pelvis (Figure 1). His past medical history was notable for von Willebrand disease, steroid-responsive immune thrombocytopenic purpura, MALT lymphoma, atrial fibrillation with a Watchman device, and a right total knee replacement complicated by chronic joint infection. He had been on suppressive amoxicillin (875mg BID) and TMP-SMX (240-1200mg BID) for approximately one year prior to presentation. His CT scan also demonstrated prostatomegaly and a 2.9 cm left renal mass, which was managed with active surveillance.

**Figure 1:**
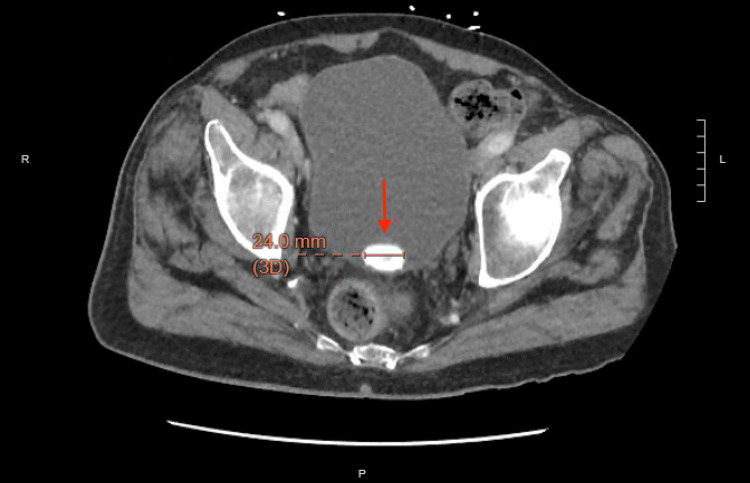
CT abdomen/pelvis obtained on ED presentation for abdominal pain Axial CT image demonstrating a 2.4 cm bladder stone (red arrow) in the distended bladder of an 80-year-old male with BPH. BPH: benign prostatic hyperplasia

Initially, the patient was hesitant to pursue surgical intervention for the bladder stone because he experienced significant blood loss requiring a 10-unit transfusion after his TURP. He was on daily tamsulosin and dutasteride and reported no urinary symptoms except for occasional mild hesitancy. Given its low Hounsfield units (426 ± 17.6 HU), the stone was thought to be composed of uric acid. The patient also had a slightly acidic urine pH (5.5-6). Medical dissolution therapy with an alkalinizing agent was attempted but was unsuccessful.

Within a year, the patient developed recurrent episodes of gross hematuria with clot passage. Repeat imaging revealed an interval increase in the size of the stone to 2.9 cm. The patient elected to undergo cystoscopy with cystolitholapaxy/laser lithotripsy and bilateral retrograde pyelograms. Preoperative care was coordinated with his hematologist, medical oncologist, and infectious disease specialist. Coagulation studies and a von Willebrand panel were checked and found to be within normal limits. Intraoperatively, the stone was found to be ovoid and mildly spiculated. It was reduced to dust with a Holmium laser, and excellent hemostasis was achieved. The patient recovered well without complication, and his hematuria resolved.

Analysis of stone fragments revealed their composition to be 100% N4-acetyl-sulfamethoxazole. The patient was advised by urology and infectious disease to switch from TMP-SMX to doxycycline, but he requested to remain on TMP-SMX given its effectiveness in preventing recurrent septic arthropathy. At the six-month follow-up, ultrasound of his kidneys and bladder showed no stones or hydronephrosis. The patient was noted to have a moderately distended, trabeculated bladder with a significant post-void volume of 323 mL, consistent with longstanding outlet obstruction due to an enlarged prostate.

Case 2

The second patient was a 70-year-old male with a history of multiple sclerosis and neurogenic detrusor overactivity complicated by recurrent UTIs. His urinary tract dysfunction was managed with a suprapubic catheter and long-term TMP-SMX (80-400 mg BID). Initially, he presented with intermittent obstruction of his suprapubic catheter and gross hematuria, which temporarily resolved with irrigation. An ultrasound was performed, which demonstrated a 1 cm echogenic bladder stone (Figure 2). He was taken to the operating room for definitive treatment with cystolitholapaxy. Cystoscopy revealed multiple small stones, which were fragmented, evacuated, and later identified as 100% N4-acetyl-sulfamethoxazole. As in the first case, this patient was advised to discontinue TMP-SMX, which he had been prescribed approximately nine months prior to presentation. Given allergies to ciprofloxacin and cephalosporins, he switched to doxycycline prophylaxis for UTIs.

**Figure 2:**
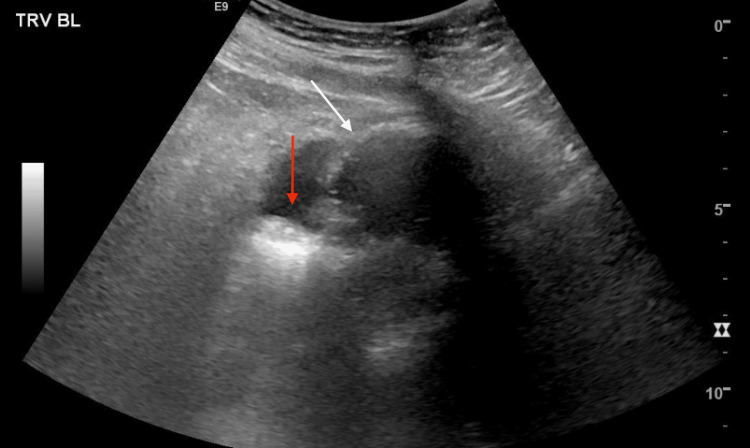
Renal bladder US obtained on ED presentation for SPT malfunction and gross hematuria Bladder ultrasound demonstrating a hyperechoic 1 cm bladder stone (red arrow) with posterior acoustic shadowing in the decompressed bladder of a 70-year-old male with a chronic suprapubic catheter (balloon marked by white arrow). SPT: suprapubic tube

## Discussion

Some side effects of TMP-SMX, including drug-induced urolithiasis, are specifically due to the sulfamethoxazole component. Like all sulfonamides, sulfamethoxazole is hepatically metabolized and excreted in the urine. Dosing is adjusted among individuals with a creatinine clearance of less than 30 mL/minute due to the potential for accumulation of metabolites, primarily N4-acetyl-sulfamethoxazole. However, compared to older sulfonamides like sulfadiazine, sulfamethoxazole is more soluble and less likely to cause crystalluria [3]. In general, N4-acetyl-sulfamethoxazole crystals precipitate in concentrated, acidic urine [4]. For this reason, co-administration of TMP-SMX and methenamine, which helps maintain low urinary pH, is contraindicated.

The first documented instance of a pure N4-acetyl-sulfamethoxazole stone occurred in a patient on TMP-SMX for bacterial prostatitis with a concomitant calcium oxalate stone [5]. Roedel et al. describe a patient with a lower pole calcium oxalate stone that quadrupled in size due to N4-acetyl-sulfamethoxazole deposition after high-dose TMP-SMX treatment for Nocardia pneumonia [6]. Both Rince et al. and Chase et al. present cases of TMP-SMX-induced urolithiasis in patients with recurrent UTIs and indwelling catheters [2,7]. Our patient with neurogenic bladder had a similar history of recurrent UTIs and a suprapubic catheter. However, our other patient was unique in that he did not have a history of urolithiasis or UTIs to suggest an abnormal urinary environment.

Urinary stasis likely contributed to bladder stone formation in both patients, especially the first patient with a history of lower urinary tract symptoms due to BPH. While the majority of case reports on TMP-SMX-induced urolithiasis describe obstructing renal stones, a 1994 review of 40 patients with sulfonamide stones found that the bladder was the most common location of the calculus [8]. Chronic inflammation and immunosuppression, such as that due to steroid-responsive ITP or multiple sclerosis, might also predispose to stone formation. DeMasi et al. propose that such factors contributed to the development of N4-acetyl-sulfamethoxazole ureteral stones in a 43-year-old man with HIV infection [9].

As in these cases, N4-acetyl-sulfamethoxazole stones are usually soft and amenable to laser therapy. Although urine alkalinization was ineffective for our patient, who had been taking high-dose TMP-SMX (400-1200 mg BID) for a year, it is unknown how long the stone had been in his bladder. Recently, Khan and Geller reported that rapid initiation of bicarbonate therapy was successful in clearing N4-acetyl-sulfamethoxazole renal stones, which developed in a 94-year-old man with a chronic Foley catheter just 9 days after starting TMP-SMX (160-800mg BID) for *Escherichia coli* bacteremia [10].

On identification of N4-acetyl-sulfamethoxazole stones, the discontinuation of TMP-SMX and transition to an alternative antibiotic like doxycycline should be strongly considered. However, if not medically reasonable or in concordance with patient preference, excellent hydration and urinary alkalinization are appropriate preventive measures. One of our patients chose to remain on TMP-SMX but continues to demonstrate incomplete voiding and will be surveyed with an ultrasound for further stone development. Given that he was asymptomatic until the bladder stone became quite large, it may be reasonable to screen similar individuals on long-term TMP-SMX.

## Conclusions

This report describes two cases of isolated N4-acetyl-sulfamethoxazole bladder stones caused by prophylactic TMP-SMX therapy. Notably, one patient had a history of BPH with storage symptoms but no prior urolithiasis or UTIs. Both patients presented with gross hematuria and required surgical treatment. We encourage urologists to consider screening, such as with routine ultrasound, for patients with factors that may promote TMP-SMX-induced urolithiasis, including incomplete bladder emptying and prolonged TMP-SMX course.
